# Priapism in Antipsychotic Drug Use: A Rare but Important Side Effect

**DOI:** 10.1155/2012/496364

**Published:** 2012-06-05

**Authors:** Igne Sinkeviciute, Rune A. Kroken, Erik Johnsen

**Affiliations:** ^1^Division of Psychiatry, Haukeland University Hospital, Sandviksleitet 1, 5035 Bergen, Norway; ^2^Section of Psychiatry, Department of Clinical Medicine, University of Bergen, Sandviksleitet 1, 5035 Bergen, Norway

## Abstract

Priapism is a rare but important side effect of antipsychotic drugs which may evolve into a urological emergency. Most antipsychotic drugs are alpha-1 adrenergic antagonists, which is thought to be the principal mechanism involved in antipsychotic-induced priapism. Other aetiologies exist, however. A case is presented with multiple episodes of priapism during the use of several different antipsychotic drugs. The case is representative of many patients treated with antipsychotic drugs, as there were hyperprolactinemia, and illicit drug use, which are known causes of priapism. Moreover, the patient used combinations of antipsychotic drugs. The case thus illustrates the etiological complexity which could delay a diagnosis of antipsychotic-induced priapism, and the problem of establishing a link between priapism and one particular ingredient of a drug combination. The case presents how a treatment regimen was finally established balancing antipsychotic efficacy to acceptable side effects and offers guidance to physicians regarding how antipsychotic-induced priapism may be resolved.

## 1. Introduction

Priapism is defined as prolonged and persistent erection of the penis without sexual stimulation [1] and is divided into a nonischemic type (arterial, high flow) that can be treated conservatively and an ischemic type (venoocclusive, low flow) which may become a urological emergency that requires immediate intervention [1]. A variety of etiological factors are implicated in the condition. Nonischemic priapism is associated with penile or perineal trauma, cocaine, metastatic malignancy, among others, whereas ischemic priapism can be caused by drugs, haematological disorders, metabolic disorders, alcohol, and other factors [2]. Drug-induced priapism is associated with antidepressants, antipsychotics, antihypertensive medications, and accounts for approximately 15% to 41% of all cases, of which antipsychotics-induced priapism is most common [3]. All antipsychotic drugs antagonize dopaminergic transmission at the dopamine type 2 (D2) receptors in the central nervous system (CNS) but are pharmacologically heterogeneous as a drug group. The drugs are usually classified into first (FGA) and second generation antipsychotics (SGAs). The FGAs are characterized by a strong affinity for the D2 receptor, whereas the SGAs are serotonergic antagonists at the 5-HT2A receptors as well as being D2 receptor antagonists [4]. In addition, most antipsychotics have affinities for a wide array of other receptors in the CNS, including histaminergic, noradrenergic, and cholinergic receptors (Table 1) [4–6].

Sexual dysfunction is prevalent in users of antipsychotics [7], and may be attributed to the effects on alpha-1 and alpha-2 adrenergic, histamine-H1, and dopaminergic receptors [8]. Sexual side effects are particularly important to recognize and address properly in populations with psychosis. These side effects are less likely to be reported spontaneously by the patient because of their sensitive nature or psychotic symptoms and are known to adversely influence adherence with medication. Reduced adherence is a major cause of psychotic relapse and rehospitalization in psychosis [9]. We present a case with repeated priapism associated with antipsychotic drug use, and how this troublesome adverse effect was eventually resolved. The patient provided informed consent. 

## 2. Case Presentation 

The case is a 33-year-old male of Hispanic ethnicity suffering from schizophrenia diagnosed during the hospitalizations reported here, with auditory hallucinations, paranoia, and negative symptoms of psychosis. He also has hepatitis C and had abused illicit drugs since the age of 17. The developmental history was normal. During a two-year period he had several admittances to psychiatric hospital because of psychotic relapses and was treated with different antipsychotic drugs. In the same period of time several episodes of priapism occurred, which gravely complicated the antipsychotic drug therapy. 

Although there were indications of priapism even earlier, the first well-documented episode of priapism occurred about 70 days after starting treatment with the SGA olanzapine. The dosage was 2.5 defined daily doses (DDDs). The basic definition of the DDD unit is the assumed average maintenance dose per day for a drug used for its main indication in adults, as developed by the World Health Organization Collaborating Centre for Drug Statistics Methodology [10]. The patient concomitantly used 0.2 DDDs of the FGA chlorprotixene. Two days prior to the priapism episode, he had illicitly used buprenorphine. The priapism resolved spontaneously. The antipsychotic drug regimen was then changed to 0.8 DDDs of another SGA, risperidone, in combination with 0.2 DDDs of chlorprotixene. A week later the patient had another episode of priapism and then a third episode after 2 more weeks. Illicit use of buprenorphine could not be ruled out in either of these instances. Urological intervention at the emergency room was necessary at both times. Risperidone was discontinued and the patient continued using 0.2 DDDs of chlorprotixene during which no episodes of priapism appeared. After 3 months the drug regimen was then changed back to olanzapine 1.5 DDDs combined with chlorprotixene and the FGA levomepromazine on demand. After a latency of about 3 weeks, priapism reoccurred. There was no illicit drug use prior to the episode. Following the last incidence of priapism the patient continued to use olanzapine for approximately 6 months without priapism being reported. The use was irregular, and in different doses. The antipsychotic medication was then briefly switched to risperidone but after 11 days changed again to the FGA perphenazine in a doses of 0.3 DDDs concomitantly with levomepromazine 0.5 DDDs. Three days later an episode of priapism occurred. Perphenazine was discontinued while treatment with levomepromazine 0.5 DDDs was continued. Five days later, there was yet another episode of priapism. All antipsychotic drugs were then discontinued for two weeks, after which he received a single dose of chlorprotixene 0.2 DDDs followed by priapism the same day. 

The patient was finally prescribed amisulpride which has a high selectivity for dopaminergic D2 and D3 receptors in the CNS and no significant affinity for adrenergic *α*-receptors [4, 11]. His mental illness responded well to the treatment and there was no priapism during the 86 days he was using the drug in doses equivalent to 2.5 DDD per day, and despite hyperprolactinemia and infrequent intake of buprenorphine in the period. The patient then chose to discontinue the drug because of muscle pains and the psychotic symptoms relapsed very quickly after discontinuation of the antipsychotic drugs. Clozapine, despite having affinity for adrenergic alpha-1 receptors was then started in increasing doses up to 1.3 DDD, administered twice daily in equal doses. Clozapine has demonstrated superior antipsychotic efficacy in treatment-refractory schizophrenia but is generally regarded as a third line antipsychotic because of its potential for inducing agranulocytosis. About 2 months after the initiation of clozapine treatment, the patient began to complain about nightly erections. The erections lasted a couple of hours, were painless but distressing to the patient. After recommendations from a urologist, the patient was prescribed 0.33 DDD of the selective beta-2 adrenoreceptor agonist terbutaline in these episodes but without any effect. The medication regimen was changed so that the patient was to take only a quarter of the daily dose of clozapine at bed time without changing the total daily dose. Moreover, one DDD of amisulpride was reintroduced concomitantly to increase the total antipsychotic drug dose and to address the negative symptoms of psychosis in particular, without increasing the adrenergic alpha-1 blockage. The psychotic symptoms were attenuated and no more occurrences of nightly erections were reported. 

In summary the patient was referred 6 times to the urological emergency room for treatment of priapism, the first 3 times by one psychiatric team, the last 3 times by another psychiatric team. The duration of priapism varied from 4 to 7 hours. In the emergency room, he was treated with penile aspiration and/or intracavernosal instillation of a sympathomimetic agent. Treatment of the last episode was more complicated, and surgical intervention was considered as the patient did not respond to the initial injections of the sympathomimetic. However, repeated injections were performed and relief of the condition was achieved. 

 Of relevance is that the patient occasionally used illicit drugs, but in the latter 4 episodes with priapism there was no temporal relationship with intake of any illicit drugs. Furthermore, serum levels of glucose and liver enzymes were within the normal boundaries. The prolactin levels were moderately elevated. 

## 3. Discussion 

The case demonstrates several important points. Priapism is a condition which could be the result of several different aetiologies. In this particular case, which is representative of many patients with psychotic disorders, there were comorbid liver disease, hyperprolactinemia, and illicit drug use. Moreover, the patient used more than one antipsychotic drug at the same time. The practice of combining antipsychotic drugs is very common but has little support in the evidence-based medicine. Antipsychotic polytherapy also increases the risk of side effects, and when side effects appear it can be difficult to disentangle which part of the combination is responsible. Antipsychotic-induced priapism seems to be associated with blockade of alpha-1 adrenergic receptors in the corpora cavernosa of the penis [3]. Andersohn and collaborators [12] found an association between the drug affinities for the alpha-1 receptor of different antipsychotics and their relative propensities for causing priapism. In most cases of drug-induced priapism no correlation is found between dosage or duration of treatment and priapism [3, 13–15]. However, there are reports of priapism in patients with elevated serum level of drug caused by CYP 450 enzyme interactions [16, 17]. In the present case, priapism occurred also at very low doses of the drugs. Furthermore, priapism appears in only a small fraction of men using medications with alpha-1 receptor-blocking properties, indicating differential sensitivity to the alpha-1 blockade [18] and/or that additional etiological factors must be present for priapism to become manifest [18, 19]. Such additional factors might involve diabetes mellitus as found in a case report of a patient using antipsychotics [20]. Also hyperprolactinemia has been implicated as a cause of priapism [21]. In the present case, the levels of liver enzymes and glucose in serum were normal but serum prolactin levels were moderately elevated. The hyperprolactinemia peaked during the use of amisulpride without any episodes of priapism, however. The case had Hispanic ethnicity. African-American men seem to be at increased risk of priapism [3] but we have not been able to find increased risk also for Hispanic men. 

The antipsychotic drugs were suspected from the start as the cause of priapism in the patient, but the initial switches made were not successful in this regard. Only after choosing an antipsychotic agent without alpha-1 receptor affinity, was the situation resolved. Amisulpride is the only registered antipsychotic drug in Norway without alpha-1 receptor affinity but another option might have been sulpiride which has a similar pharmacological profile [6]. Unfortunately, other side effects emerged leading to discontinuation of amisulpride followed by a severe relapse of psychotic symptoms. In the treatment-refractory patient clozapine is the drug of choice but this drug also induced distressing erections in this patient. The final solution was to combine a moderate dose of clozapine with a moderate dose of amisulpride. In this particular case sufficient antipsychotic efficacy was obtained while at the same time the tolerability was satisfactory to the patient because the drugs have distinctly different side effect profiles. Theoretically, each drug contributed to the antipsychotic efficacy, whereas no additive effect occurred with regards to side effects. 

Lastly, the present case may also indicate a gradual lowering of the threshold for priapism as the latter incidences occurred only after a few days of drug use, or after a single dose. The same finding is reported in a case using quetiapine [14]. Hypothetically this could by caused be increased alpha-1 receptor sensitivity, but studies with prospective designs are needed to further elaborate this observation. 

## Figures and Tables

**Table 1:** The relative affinities for selected receptors of some antipsychotic drugs.

Rec	Chlorpromazine	Haloperidone	Amisulpride	Sulpiride	Clozapine	Risperidone	Olanzapine	Quetiapine
D1	+	++	—	—	++	+	++	+
D2	+++	++++	+++	+++	+	+++	++	+
D3	+++	+++	+++	+++	+	+++	++	+
D4	++	+++	—	—	++	+++	++	++
5HT1A	—	—	—	—	+	+	—	+
5HT2A	+++	++	—	—	++	++++	+++	++
5HT2C	++	—	—	—	++	++	++	—
*α*1	++++	+++	—	—	+++	++++	++	+++
H1	++++	++	—	—	+++	++	+++	++
M1	++	—	—	—	+++	—	+++	+

Notes: Rec: receptor; D1–D4: dopamine receptors 1–4; 5-HT1A–2C: serotonine receptors 1A–2C; *α*1: alpha-1 adrenergic receptor; H1 = histamine receptor 1; M1: muscarine receptor 1. Adapted from Abi-Dargham & Laurelle 2005 [4]; Roth et al. 2004 [5]; Miyamoto et al 2005 [6].
